# Urinary Alpha-1-Acid Glycoprotein Is a Sensitive Marker of Glomerular Protein Leakage at Altitude

**DOI:** 10.1089/ham.2018.0017

**Published:** 2018-09-18

**Authors:** Ben J. Talks, Susie B. Bradwell, John Delamere, Will Rayner, Alex Clarke, Chris T. Lewis, Owen D. Thomas, Arthur R. Bradwell

**Affiliations:** ^1^ Medical School, College of Medical and Dental Sciences, University of Birmingham, Birmingham, United Kingdom.; ^2^ Birmingham Medical Research Expeditionary Society, Birmingham, United Kingdom.; ^3^ East Surrey Hospital Redhill, Surrey, United Kingdom.; ^4^ The Binding Site Group Ltd., Birmingham, United Kingdom.; ^5^ Newcastle Upon Tyne Hospitals NHS Foundation Trust, Newcastle Upon Tyne, United Kingdom.; ^6^ Raigmore Hospital, Inverness, Highland, United Kingdom.; ^7^ Taunton and Somerset NHS Foundation Trust, Taunton, United Kingdom.

**Keywords:** alpha-1-acid glycoprotein, altitude, glomerular capillary wall, orosomucoid, proteinuria

## Abstract

Talks, Ben J., Susie B. Bradwell, John Delamere, Will Rayner, Alex Clarke, Chris T. Lewis, Owen D. Thomas, and Arthur R. Bradwell. Urinary alpha-1-acid glycoprotein is a sensitive marker of glomerular protein leakage at altitude. *High Alt Med Biol*. 19:295–298, 2018.—Proteinuria is an established feature of ascent to altitude and may be caused by a loss of negative charges on glomerular capillary walls (GCWs). To test this hypothesis, we measured two similar sized but oppositely charged proteins in urine: negatively charged alpha-1-acid glycoprotein (α1-AGP, 41–43 kDa) and positively charged dimeric lambda free light chains (λ-FLCs, 50 kDa). Twenty-four-hour urinary leakage was compared with albumin, a 66 kDa negatively charged protein. We studied 23 individuals (ages 23–78 years, male = 17) at baseline (140 m) and daily during an expedition to 5035 m. The results showed a significant increase in median urinary leakage of α1-AGP (*p* < 0.0001; 6.85-fold) and albumin (*p* = 0.0006; 1.65-fold) with ascent to altitude, but no significant increase in leakage of λ-FLCs (*p* = 0.39; 1.14-fold). α1-AGP correlated with the daily ascent profile (*p* = 0.0026) and partial pressure of oxygen (*p* = 0.01), whereas albumin showed no correlation (*p* = 0.19). Urinary α1-AGP was a more sensitive marker of altitude proteinuria than urinary albumin and λ-FLCs, and supported the possibility of loss of GCW negative charges at altitude.

## Introduction

There is a well-established association between ascent to high altitude and urinary albumin excretion, which correlates with hypoxia and symptoms of acute mountain sickness (AMS) (Rennie and Joseph, 1970; Pines, 1979; Bradwell and Delamere, 1982).

Proteinuria involves the passage of protein from the capillary lumen into Bowman's space across the three layers of the glomerular capillary wall (GCW): the glomerular endothelial cells, glomerular basement membrane, and podocytes. Each of these layers has a negative charge and also filters by molecular size (Maack et al., 1979). Proteinuria results from two different mechanisms: (1) increased permeability of the GCW and (2) failure of proximal tubular reabsorption (Jefferson et al., 2008). Permeability of the GCW depends on molecular size, molecular charge, and hydrostatic pressure in the glomerulus (Jefferson et al., 2008). Albumin has been used to assess urinary protein leakage at high altitude. It is a 66 kDa negatively charged protein (pI-4.7) (Christensen et al., 2012), which is similar in size to the maximum pore diameter of the slit diaphragm of the GCW (Jarad and Miner, 2009). Investigation of glomerular charge selectivity at altitude has also been undertaken using radioactively labeled charged dextran molecules (Brenner et al., 1978; Winterborn et al., 1990), and suggests that GCW negative charge decreases at altitude. Failure of proximal tubular reabsorption was shown to make a small contribution to altitude proteinuria in a study using lysine to inhibit proximal tubular protein uptake (Winterborn et al., 1987).

Our present study uses a different approach, namely the urinary leakage of two similar size but oppositely charged serum proteins: negatively charged alpha-1-acid glycoprotein (α1-AGP: pI-2.7) and positively charged lambda free light chains (λ-FLCs: pI ∼5.7). α1-AGP is a 41–43 kDa acute phase protein, largely synthesized by the liver (Fournier et al., 2000). Owing to its smaller size, the renal filtration of α1-AGP is less size restricted than albumin. λ-FLCs are produced by immune cells of plasma cell lineage that predominantly exist as 50 kDa dimers (Bradwell, 2010). Both these molecules are similar in size to charged dextran molecules previously studied (Brenner et al., 1978).

We hypothesized that urinary leakage of α1-AGP may better reflect changes in GCW charge and provide a more sensitive marker of glomerular protein leakage at altitude than albumin and second that increases in the excretion of α1-AGP relative to λ-FLCs would support a reduction in GCW negative charge.

## Methods

### Individuals and sample collection

Twenty-three individuals (ages 23–78 years, male = 17) were studied before and during an altitude research expedition to 5035 m (Chimborazo, Ecuador). Participants were members of the Birmingham Medical Research Expeditionary Society. After a 3-day acclimatization period at 2800 m in Quito, participants ascended in stages by bus and gentle walking to 5035 m (Fig. 2). In addition, there were three acclimatization walks of 2 to 3 hours each, at moderate exercise intensity (Fig. 2). One individual had to descend early because of AMS on day 8 of the expedition, prematurely stopping their sample collection.

At baseline (140 m) and on each day of the expedition, 24-hour urine samples were collected into plastic bottles containing 0.1% sodium azide as preservative. Two milliliters aliquots were taken from each bottle and stored in polystyrene containers with solid carbon dioxide (−78.5°C) and subsequently at −80°C in the UK until analysis. Venous blood samples were taken daily, centrifuged, and the sera were stored alongside urine samples. Daily arterialized ear lobe partial pressure of oxygen (pO_2_) measurements were obtained using an Abbott i-STAT (Lewis et al., 2018), and twice daily Lake Louise (LL) AMS scores were recorded.

The three proteins were measured by immunoassays produced by the Binding Site Ltd. (Birmingham, UK). For the serum analysis of α1-AGP, we used automated immunoturbidimetry (Optilite™; lower limit of detection, 0.19 g/L). Urine measurements were carried out by automated immunoturbidimetry for albumin (SPA™; lower limit of detection, 11 mg/L) and λ-FLCs (BNII™; lower limit of detection, 0.05 mg/L), whereas α1-AGP was measured using radial immunodiffusion (lower limit of detection, 0.12 mg/L).

### Statistical analyses

Statistical analysis was performed using GraphPad Prism 7. All hypothesis tests were two-tailed; significance level was established at the level of α <0.05, with Bonferroni correction for multiple comparisons of daily protein leakage. Results are presented as median ± interquartile range (IQR). A Mann–Whitney U test was used to compare 24-hour urine leakage of λ-FLCs between baseline and ascent to 5035 m on day 8 of the expedition. A Kruskal–Wallis test with Dunn's testing for multiple comparisons was used for comparing daily protein leakage of α1-AGP and albumin to baseline throughout the expedition. Daily protein leakage was considered significant if α <0.05/10 = 0.005. Spearman's rank correlation tests were performed to look for associations of urinary protein leakage with altitude, degree of hypoxia, and AMS.

### Ethics approval

The study was approved by Chichester University Research Ethics Committee (Protocol No.: 1314_42) and was performed according to the Declaration of Helsinki. All individuals provided signed informed consent.

## Results

**FIG. 1.**
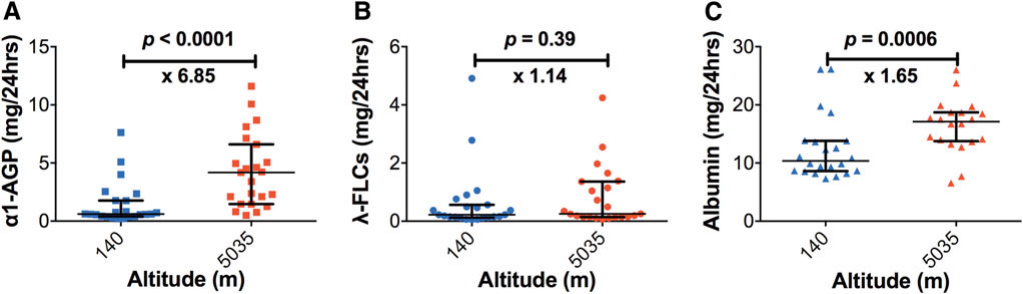
Difference in 24-hour urinary excretion of **(A)** α1-AGP, **(B)** λ-FLCs, and **(C)** albumin measured at baseline (140 m) and altitude (5035 m) displayed as median ± IQR. *Squares*, *circles*, and *triangles* indicate leakage in mg/24 hrs of α1-AGP, λ-FLCs, and albumin, respectively. α1-AGP, alpha-1-acid glycoprotein; λ-FLCs, lambda free light chains; IQR, interquartile range.

Figure 1 shows the urinary leakage of α1-AGP, λ-FLCs, and albumin measured in 24-hour urine samples at baseline (140 m) and at the day of ascent to 5035 m. There was a significant increase in median urinary leakage of α1-AGP (0.61 ± 0.37 mg/24 hours at 140 m vs. 4.18 ± 5.14 mg/24 hours at 5035 m; 6.85-fold; *p* < 0.0001) and albumin (10.37 ± 5.2 mg/24 hours at 140 m vs. 17.12 ± 4.97 mg/24 hours at 5035 m; 1.65-fold; *p* = 0.0006). There was no significant change in median urinary leakage of λ-FLCs (0.22 ± 0.44 mg/24 hours at 140 m vs. 0.25 ± 0.22 mg/24 hours at 5035 m; 1.14-fold; *p* = 0.39).

There was no significant increase in α1-AGP concentrations in the sera at altitude (Mann–Whitney U test; *p* = 0.085), and no association between urine and serum concentrations of α1-AGP (Spearman's ρ, *r*_s_ = 0.36, *p* = 0.13).

**FIG. 2.**
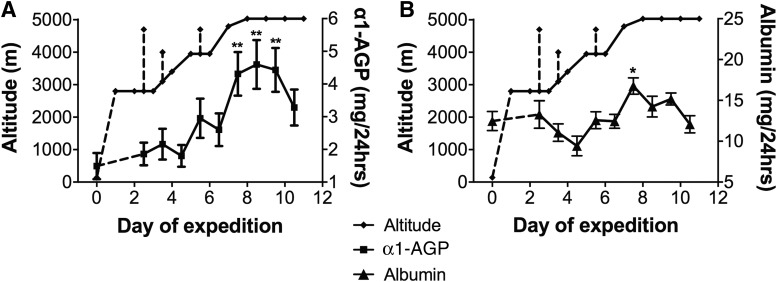
Urine excretion of **(A)** α1-AGP and **(B)** albumin in daily 24-hour urine samples alongside daily altitudes (median ± IQR; *p < 0.005; **p < 0.0001). Moderate intensity acclimatization hikes of 2 to 3 hours duration are shown as *dashed vertical lines*.

**FIG. 3.**
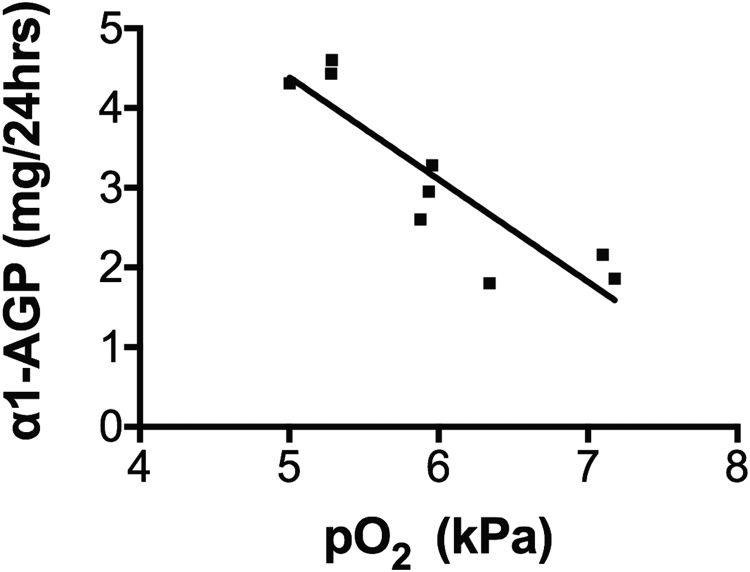
Mean oxygen concentrations in arterialized ear lobe samples (pO_2_) on 9 days of the expedition versus mean 24-hour urine α1-AGP excretion (*p* = 0.01, *r* = −0.82). *Squares* indicate an α1-AGP leakage in mg/24 hrs. pO_2_, partial pressure of oxygen.

Twenty-four-hour urine excretion of α1-AGP and albumin is shown alongside daily altitudes in Figure 2. α1-AGP increases (*p* = 0.0026, *r*^2^ = 0.89) but not albumin increases (*p* = 0.19, *r*^2^ = 0.49) correlated with daily altitudes over the 8-day ascent. There was a significant correlation between blood oxygen concentrations (pO_2_) over the 9 days of the expedition and mean 24-hour urine excretion of α1-AGP (*p* = 0.01. *r* = −0.82) (Fig. 3).

There was a nonsignificant correlation between α1-AGP excretion and the day of maximum LL AMS scores for individuals (median = 4; IQR = 3; *p* = 0.06; *r* = −0.42). There was no association between daily excretion of either α1-AGP or albumin and daily AMS scores.

**FIG. 4.**
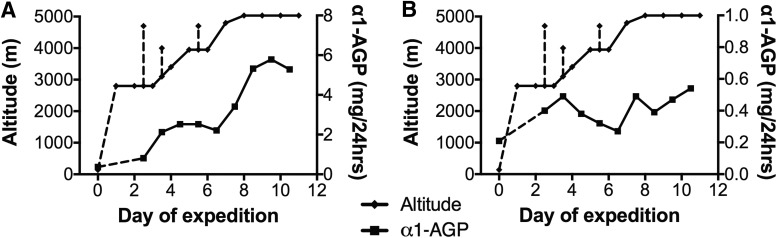
α1-AGP measured in serial 24-hour urine samples daily altitude for two individuals **(A, B)**. Moderate intensity acclimatization hikes of 2 to 3 hours duration are shown as *dashed vertical lines*.

The magnitude of α1-AGP excretion varied between individuals (Fig. 1), as did its association with altitude (Fig. 4). This was not associated with individuals' age, gender, degree of hypoxia, acclimatization experience, or LL AMS scores.

## Discussion

There was a significant increase in the urinary excretion of negatively charged α1-AGP and albumin with ascent to high altitude but not positively charged λ-FLCs (Fig. 1). This demonstrates a differential leakage of charged proteins. The percentage increase in α1-AGP was greater than albumin, indicating it was a more sensitive marker of glomerular permeability. This supports the fact that albumin is closer to the maximum pore size of the podocyte slit diaphragm.

The increase in α1-AGP correlated better with ascent to altitude than albumin (Fig. 2). Minor dips in α1-AGP excretion during rest days at constant altitudes may reflect glomerular charge recovery. On an individual basis, there was considerable variation in α1-AGP excretion (Fig. 4) that was not related to age or LL AMS symptom scores. We excluded increases in serum α1-AGP as a cause of increased urinary excretion by demonstrating no change in serum concentrations with increases in altitude.

The proportion of urinary protein excretion attributable to glomerular filtration or failure of tubular reabsorption mechanisms cannot be assessed from our results. However, it should be noted that there is a highly efficient specific reabsorption for albumin through FcRn receptors (Sand et al., 2015). In contrast, α1-AGP and λ-FLCs are reabsorbed by the megalin/cubilin pathway that operates for all proteins passing through renal tubules (Christensen and Birn, 2002).

These results add to the previously suggested loss of glomerular charge using radioactively labeled charged dextran molecules (Winterborn et al., 1990). By using endogenous proteins, we avoided the administration of radioactive isotypes and potentially toxic dextran molecules (Walton, 1954).

The mechanism for the proposed loss of glomerular negative charge is unknown. However, protein excretion is associated with increasing hypoxia (Fig. 3) and may have a similar cause to that observed during hard exercise (Poortmans and Vanderstraeten, 1994).

### Limitations of our study

This study involved 23 individuals ascending to high altitude. The relatively small sample size and modest rate of ascent may explain the lack of association of α1-AGP excretion with age, degree of hypoxia, or LL AMS symptom scores.

Our analysis of urinary protein excretion was based upon 24-hour urine samples that were collected by an experienced medical team. This is impractical for tourist trekking groups. Studies of early morning urine and random urine samples are required to validate our observation in a wider setting. We cannot comment on the relationships between increased α1-AGP excretion and AMS, since there was minimal illness in the study. Blood pressure, respiratory rate, and hematocrit were not measured.

## Conclusion

There was a greater increase in urinary excretion of negatively charged albumin and α1-AGP than positively charged λ-FLCs with ascent to altitude. This can be attributed to loss of GCW charge. Furthermore, α1-AGP showed greater changes than albumin with altitude exposure, indicating it is a more sensitive marker.
